# Obesity-Induced Adipose Tissue Inflammation as a Strong Promotional Factor for Pancreatic Ductal Adenocarcinoma

**DOI:** 10.3390/cells8070673

**Published:** 2019-07-03

**Authors:** Hui-Hua Chang, Guido Eibl

**Affiliations:** Department of Surgery, David Geffen School of Medicine at UCLA, Los Angeles, CA 90095, USA

**Keywords:** obesity, adipose tissue inflammation, pancreatic ductal adenocarcinoma

## Abstract

Pancreatic ductal adenocarcinoma (PDAC) is expected to soon become the second leading cause of cancer related deaths in the United States. This may be due to the rising obesity prevalence, which is a recognized risk factor for PDAC. There is great interest in deciphering the underlying driving mechanisms of the obesity–PDAC link. Visceral adiposity has a strong correlation to certain metabolic diseases and gastrointestinal cancers, including PDAC. In fact, our own data strongly suggest that visceral adipose tissue inflammation is a strong promoter for PDAC growth and progression in a genetically engineered mouse model of PDAC and diet-induced obesity. In this review, we will discuss the relationship between obesity-associated adipose tissue inflammation and PDAC development, with a focus on the key molecular and cellular components in the dysfunctional visceral adipose tissue, which provides a tumor permissive environment.

## 1. Obesity and Pancreatic Ductal Adenocarcinoma

Pancreatic cancer, of which pancreatic ductal adenocarcinoma (PDAC) accounts for the vast majority, is an aggressive disease with an overall 5-year survival rate of ~8% [1]. Currently, as the third leading cause of cancer mortality in both men and women, pancreatic cancer is expected to soon become the second leading cause of cancer-related deaths in the United States [2]. One of the recognized risk factors for PDAC and several other gastrointestinal cancers is obesity [3,4,5,6,7,8]. A recent analysis by the National Institutes of Health reported that 16.9% of all PDAC cases in the Unites States are estimated to be attributable to excess body weight (in comparison, the proportion of PDAC cases attributable to cigarette smoking was 10.2%) [9]. Besides promoting tumorigenesis, obesity might also complicate the treatment of established cancers [10], not only by altering the pharmacokinetics and pharmacodynamics of administered anti-cancer drugs [11], but also by modulating the tumor microenvironment (TME) [12]. Notably, worse prognosis is reported in patients who are either overweight or obese at the time of PDAC diagnosis [13,14]. Considering the current obesity pandemic, there is great interest in deciphering the underlying biological basis of this compelling epidemiological link.

The mechanisms underlying the obesity–cancer link are likely multifactorial, with metabolic perturbations and inflammation being the current research focus. Other mechanisms, such as alterations in the gut microbiome, changes in gastrointestinal peptides, and sex hormones, certainly play an important role as well. For example, obesity-associated insulin resistance and hormonal changes (e.g., compensatory hyperinsulinemia and elevated insulin-like growth factors (IGFs)) are among the various driving mechanisms postulated to explain the obesity–cancer link [15]. In accordance with this notion, type 2 diabetes, hyperinsulinemia, and increased circulating IGF-1 are established risk factors for pancreatic and other types of cancers [16,17,18,19,20]. In addition to the insulin/IGF axis, increasing attention has focused on adipose tissue (AT) dysfunction characterized by a pro-inflammatory state, which can contribute not only to the pathogenesis of insulin resistance [21] but also to the development and promotion of cancer. Utilizing a genetically engineered mouse model of PDAC and diet-induced obesity, our own data suggest that inflammatory responses in the AT surrounding the pancreas is a strong promotional factor for PDAC growth and progression [22]. AT inflammation associated with increased abdominal adiposity, which involves various cellular and extracellular components (e.g., infiltrated macrophages and cytokines), can shape the peri-tumoral micro-environment favorable for PDAC development, both locally and systemically. More detailed cellular and molecular characteristics of dysfunctional and inflamed AT associated with the obese state, and their roles in tumor development, will be discussed in the later sections.

## 2. The Role of Adipose Tissue Inflammation in Obesity-Promoted Tumor Development

### 2.1. Classification and Physiological Roles of AT

White adipose tissue (WAT), the most abundant type of AT in adult humans, stores excess energy in the form of triglycerides, which can be mobilized via lipolysis to release fatty acids for usage by other tissues. Based on anatomical locations, AT can also be organized into different types of depots: Subcutaneous and visceral. These depots are heterogeneous with regard to their morphological, molecular, and metabolic profiles [23], and exhibit major gender differences. Visceral adipose tissue (VAT), which encompasses omental, mesenteric, and other intra-abdominal fat pads, is considered of great relevance in obesity-related comorbidities, as increased VAT area is more closely associated with metabolic dysfunction and cancer than subcutaneous fat [7,23,24,25]. Surgical removal of VAT prior to high fat diet feeding is shown to prevent obesity-induced insulin resistance in mice [26]. Since VAT is in close proximity to digestive organs, including the pancreas, and is the primary source of systemic and local chronic inflammation during obesity [27], it is particularly pertinent to the topics covered in this review. For example, VAT (but not subcutaneous fat) and pancreatic fatty infiltration are significantly correlated with pancreatic intraepithelial neoplasia (PanIN), the precursor lesions of PDAC [28]. Interestingly, the increased PDAC incidence in obese conditional Kras^G12D^ (KC) mice was largely observed in male animals [29], which correlated with a greater expansion of VAT in obese male mice, as compared to obese female mice that preferentially gained subcutaneous fat [22].

Besides being a key regulator of energy homeostasis, AT is regarded as an important endocrine organ secreting various factors collectively known as “adipokines”, and growingly recognized as a prominent immune site harboring macrophages and other types of immune cells. Overall, this dynamic organ is composed of mature adipocytes and the “stromal-vascular fraction” comprising a mixture of mesenchymal, hematopoietic, and endothelial cell types. Under homeostatic conditions, the cells with diverse functional roles act cooperatively to support the metabolic and physiological functions of the organ. Specifically, healthy AT maintains an anti-inflammatory phenotype characterized by the secretion of anti-inflammatory adipocytokines (e.g., adiponectin) and enrichment of regulatory immune cells, such as M2 macrophages, eosinophils, Th2 leukocytes, and myeloid-derived suppressor cells that limit AT inflammation. In obesity, on the other hand, the regulatory anti-inflammatory network in AT is disrupted and skewed to a pro-inflammatory state, with substantial changes in adipokines and the number and function of AT immune cells (see next section), leading to sustained chronic inflammation.

### 2.2. Obesity-Associated AT Inflammation

Chronic over-nutrition is manifested as adipose tissue expansion via hyperplasia (increase in adipocyte numbers) and/or hypertrophy (enlargement of existing adipocytes). Adipocyte hyperplasia, which occurs more readily in subcutaneous depots than in VAT, is normally associated with no/weak inflammation and improved insulin sensitivity and therefore regarded as a “metabolically healthy” expansion of AT. Conversely, visceral adipocyte hypertrophy, which is often associated with cellular stress, pro-inflammatory responses, and impaired insulin sensitivity, is recognized as unhealthy pathological expansion and an important contributor to the obesity-related metabolic disorders [30]. When hypertrophic adipocytes are overloaded with lipids, they become hypoxic and undergo stress responses and cell death, which are correlated with a pro-inflammatory secretome, increased pro-inflammatory immune cell infiltration, and ectopic lipid deposition in other metabolically active organs [31], each of which may contribute to progressive insulin resistance and the pathogenesis of type 2 diabetes.

Adipocyte hypertrophy is associated with a shift in the adipokine secretion profile [32]. Specifically, there is a significant increase in the production of pro-inflammatory factors, such as leptin, tumor necrosis factor-α (TNF-α), interleukin 6 (IL-6), monocyte chemoattractive protein-1 (MCP-1), and plasminogen activator inhibitor-1 (PAI-1). On the contrary, the release of adiponectin, a major anti-inflammatory adipokine, is reduced with increased adiposity in response to AT inflammation [33]. These adipocyte-derived mediators, which link adipocytes with adipose-resident immune cells, lead to an altered composition of the adipose stromal-vascular fraction characterized by reduced numbers of anti-inflammatory cells (e.g., eosinophils, T-regs, Th2 cells) and an increased recruitment of pro-inflammatory Th1 cells, neutrophils, IFNγ-producing natural killer (NK) cells, and monocytes/macrophages [34,35].

The detailed landscape of immune cell changes in AT during obesity is extremely complex and virtually every immune cell type plays a role in obesity-associated metabolic diseases [36]. However, the most prominent feature is monocyte infiltration and differentiation into pro-inflammatory macrophages, which surround necrotic adipocytes and form the histological hallmark of AT inflammation called “crown-like structures” [37]. AT macrophages (ATM), which can account for up to 50% of the cellular content in the obese AT, are the primary source of pro-inflammatory cytokines (e.g., IL-6, TNF-α, IL1-β), and have been shown to be centrally important in obesity-related metabolic dysfunction [38,39,40,41]. The increase in ATM abundance during obesity is crucial for exacerbating the inflammatory response and is mainly caused by the recruitment of circulating bone marrow-derived monocytes through an MCP-1 (CCL2)/CCR2- dependent mechanism [42,43,44]. While resident ATMs in the lean state display an anti-inflammatory M2-polarized phenotype, obesity-associated ATM polarize towards the pro-inflammatory M1 state in response to the disturbed immune and cytokine profile and other environmental cues [35]. Interestingly, the oversimplified M1/M2 dichotomy is revised by the recent discovery of the “metabolically-activated” type ATM, which is metabolically activated by excessive saturated free fatty acids (FFAs), hyperinsulinemia, and hyperglycemia and is functionally distinct from the classical M1 macrophage activated by bacterial challenge [45]. As mentioned above, it is well established that AT inflammation and ATMs play a key role in the pathogenesis of metabolic syndromes, mainly via secreted mediators. However, much less is known about their role in cancer, especially PDAC.

Besides intrinsic danger signals, exogenous factors, such as gut microbiota-derived pathogen-associated molecular patterns (PAMPs), are also implicated in the reprogramming of immune cells toward pro-inflammatory subtypes in obese AT. Diet-induced obesity is associated with a robust change in gut microbiota composition [46,47] and impaired intestinal barrier integrity, correlating with an increased expression of pro-inflammatory cytokines in the AT [48,49]. Induced by gut dysbiosis and leaky intestinal tight junctions, circulating levels of lipopolysaccharide (LPS; a cell wall component of gram-negative bacteria) are found to be 2 to 3-fold higher in obese humans and rodents as compared to their lean counterparts, a phenomenon known as “metabolic endotoxemia” [50,51]. Elevated LPS levels in tissues, e.g., AT, can stimulate the innate immune response by activating Toll-like receptor 4 (TLR4) on macrophages, leading to the induction of NF-κB and pro-inflammatory cytokine production. Using germ-free mouse models, microbiota-derived LPS has been shown to promote macrophage infiltration and M1 polarization in WAT, independent of its role in regulating fat mass and glucose metabolism [52]. Additionally, mice fed a diet rich in saturated lipids (lard) exhibited reduced insulin sensitivity, enhanced WAT inflammation, and increased activation of TLRs compared with mice fed a fish oil diet, and such phenotypic differences were in part attributable to differences in gut microbiota composition [53]. These authors further demonstrated that lard-induced ATM accumulation was mediated by adipocyte-produced MCP-1, which was induced by microbial-derived factors through activation of TLR4 and the downstream adaptor molecules, MyD88 and TIR-domain-containing adapter-inducing interferon-β (TRIF) [53]. Together, these data strongly suggest that intestinal microbiota contribute to changes in immune cell function and AT inflammation during diet-induced obesity.

### 2.3. AT Inflammation in PDAC Development: The Molecular Links

Inflammation has long been associated with the development of cancer, including PDAC [54,55]. In a mouse model of obesity-promoted Kras^G12D^-driven pancreatic neoplasia, with obesity induced by a high-fat high-calorie diet (HFCD), we observed a higher percentage of advanced PanIN lesions [56] and increased PDAC incidence [29], accompanied by prominent signs of inflammatory immune cell infiltration and elevation of pro-inflammatory cytokines in the pancreas [56] as well as the peri-pancreatic AT [22]. In addition, deficiency in hormone-sensitive lipase (HSL) was associated with AT inflammation and an acceleration of pancreatic cancer development in conditional Kras^G12D^ mice [57]. Cumulating evidence suggest a model in which diet-induced obesity leads to a robust inflammatory response in visceral peri-pancreatic (or intra-pancreatic [28]) adipose tissue, which may in turn promote inflammation and neoplastic progression in the neighboring pancreas, via soluble factors secreted from adipocytes and/or adipose-infiltrating immune cells.

#### 2.3.1. Leptin, Adiponectin, and Other Adipokines

Obese individuals are characterized by dramatic changes in adipokine production, and several of these adipokine changes are associated with cancer development. The most prominent examples are the increased levels of the pro-inflammatory leptin [58,59] and the reduced levels of the anti-inflammatory adiponectin [60,61]. In several cancer models, leptin has been shown to promote cell proliferation, migration, and invasion through activation of the Janus Kinase/Signal Transducer and Activator of Transcription (JAK/STAT) pathway and the subsequent oncogenic phosphatidylinositol 3-kinase (PI3K)/Akt and extracellular signal-regulated kinase (ERK) signaling, leading to increased expression of inflammatory cytokines, angiogenic factors, and apoptotic proteins [61,62,63,64]. Adiponectin, on the other hand, seems to exert antagonistic effects on tumor growth and progression through activation of AMPK and protein tyrosine phosphatase 1B (PTP1B) [62,65]. However, the roles of circulating leptin and adiponectin in PDAC development remain debatable [66], as some studies found higher adiponectin/leptin ratios associated with pancreatic cancer [67,68]. Also, adiponectin transcription is activated by nuclear receptor 5A2 (NR5A2), an important risk factor identified by a genome-wide association study (GWAS) for pancreatic cancer [69]. Further, in our recent study using HSL deficient conditional Kras^G12D^ mice, decreased plasma leptin levels were observed in animals with advanced PDAC [57], suggesting that leptin may not play a central mechanistic role in PDAC promoted by AT inflammation. Besides systemic effects, adipokines generated by adjacent adipose tissue might act locally directly on tumor cells [70,71] or modulate inflammation and immunity in the pancreatic TME [72,73]. In that regard, it is noteworthy that lipocalin 2, a novel adipokine elevated in obesity subjects [74], has been shown to promote obesity-associated PDAC by stimulating the pro-inflammatory response in the TME [75].

#### 2.3.2. Pro-Inflammatory Cytokines and Chemokines

Although the events initiating obesity-associated AT inflammation are not completely understood, increased pro-inflammatory factors produced by adipocytes and infiltrating ATMs or other immune cells in the dysfunctional AT create a systemic and local inflammatory environment. Some of these factors elevated in obese subjects, such as tumor-promoting cytokines (e.g., TNF-α and IL-6) and monocyte chemo-attractants (e.g., MCP-1 [76,77]), are implicated in PDAC development and progression.

Increased expression of TNF-α is observed in obese AT [40,78], where this key pro-inflammatory cytokine is thought to act locally as an autocrine or paracrine cytokine, since systemic TNF-α levels do not necessarily reflect local changes in TNF-α concentrations [78,79]. A central role for TNF-α in obesity-associated early pancreatic tumor promotion has been demonstrated by genetic deletion of tumor necrosis factor receptor 1A (*TNFR1*) [80]. Functionally, TNF-α activates JNK and NF-κB pathways, which are known to promote cell proliferation, invasion, and metastasis in various cancers, including human PDAC, where NF-κB is constitutively activated [81,82,83]. NF-κB activation by TNF-α can crosstalk with Notch signaling, which cooperates with oncogenic KRAS to promote PDAC progression [84]. In addition, activated NF-κB can stimulate the production of chemokines (such as MCP-1), by adipocytes and pre-adipocytes, leading to increased infiltration of pro-inflammatory macrophages [85,86]. Further, NF-κB is a critical transcription factor of M1 macrophages, regulating the expression of a variety of inflammatory genes, including those encoding IL-6, IL-1β, MCP-1, and cyclooxygenase-2 (COX-2), shown to play important roles in obesity-related PDAC promotion [55]. Adipose expression of IL-6, another key modulator in inflammation-associated tumorigenesis [87,88], is also upregulated in obese individuals [40,79]. IL-6 exerts its pro-tumorigenic and pro-invasive activities through the activation of the STAT3 signaling pathway, which is essential for PDAC development in the Kras-driven mouse model [89,90,91] and observed in human PDAC [92,93]. The effects of IL-1 are similar to those of TNF-α, and IL1β gene promoter single nucleotide polymorphisms (SNPs) are linked with pancreatic cancer risk [94]. These key cytokines, whose production is increased with obesity, also play critical roles in insulin resistance [78,95], adipokine regulation [96], and tumor stroma modulation [12,97,98], indirectly influencing tumor cell growth. Importantly, these inflammatory cytokines are associated with a poor prognosis in PDAC [99,100,101]. Overall, PDAC progression is characterized by convergent activation of inflammatory transcriptional factors (i.e., NF-κB and STAT3) [102], which can be stimulated by the pro-inflammatory cytokines whose secretion is markedly enhanced in dysfunctional AT with increased adiposity (especially visceral adiposity).

#### 2.3.3. Microbiota and Obesity-Promoted PDAC

The gut microbiome could be closely related to obesity-associated chronic inflammation though TLR activation in the immune cell compartment, amplifying inflammatory responses and cytokine production that can fuel tumor growth. Although the effects of gut microbiota on tumorigenesis are mostly highlighted in other inflammation-driven, obesity-related gastrointestinal malignancies (e.g., colon and liver cancer) [103], it is well-documented that dysbiosis of oral bacteria is associated with increased risk for pancreatic cancer [104,105,106]. In experimental mouse models, LPS and TLR4 activation could enhance the severity of acute pancreatitis [107,108]. Also, ligation of TLRs, possibly through NF-κB and MAP kinase pathways, are shown to exacerbate pancreatic fibro-inflammation and accelerate Kras-driven pancreatic tumorigenesis [109,110,111]. However, less is known regarding how obesity-induced gut dysbiosis and AT inflammation contribute to PDAC development. Based on evidence linking the microbiome and inflammation-associated PDAC, and the notion that metabolic endotoxemia can be a strong promoter of ATM infiltration and M1 polarization, gut bacteria are likely to play a role in the interface of obesity, AT inflammation, and PDAC. Regardless, these interactions need to be further tested in the context of obesity.

#### 2.3.4. Other Factors Associated with AT Dysfunction in Obesity

Obesity and the inflammatory environment are typically associated with oxidative stress, another important feature of dysfunctional AT, conferring genetic instability that can promote the acquisition of oncogenic mutations in neighboring pancreatic cells [112,113]. The elevated oxidative stress can result from enriched reactive oxygen species (ROS) and reactive nitrogen intermediates (RNIs) generated by inflammatory cells, as well as mitochondrial dysfunction and lipid oxidation related to increased release of surplus free fatty acids (FFA) and ectopic fat deposition. Interestingly, in our previous study involving a mouse model of obesity-promoted PDAC, exome sequencing of advanced pancreatic intraepithelial neoplasia (PanIN) lesions identified numerous genetic variants unique to the HFCD (and diet-induced obesity) group [29]. These genetic alterations are found in genes involved in oncogenic pathways that are commonly implied in PDAC, including the insulin and PI3K/Akt pathway.

## 3. Interventional Perspectives

Based on the increasingly recognized link between obesity-induced AT inflammation and PDAC development, AT inflammation has become an intriguing target for PDAC interception. There are several possibilities to disrupt the promoting effects of (obesity-induced) AT inflammation on PDAC growth and progression. Besides general health-promoting strategies aimed at reducing obesity (e.g., weight reduction), which certainly will positively impact AT inflammation, a detailed understanding of the mechanistic link between AT inflammation and PDAC will lead to targeted approaches. Inhibiting major pro-inflammatory mediators secreted by inflamed AT or blocking cytokine receptors in the pancreas might be a promising approach. For example, anti-TNF-α antibodies and antagonists of IL-1 and IL6 receptors exhibit anti-tumor effects in pre-clinical models of PDAC [101,114,115,116,117]. However, the blockade of these cytokines, although proven to be effective in treating other inflammatory diseases, has had limited success in patients of metabolic diseases [118,119] or PDAC [120,121]. Given the complexity of the immune cell changes and inflammatory responses in the AT during obesity, with the multitude of inflammatory mediators, targeting single or even few cytokines simultaneously might not be the best strategy. Blocking major pathways driving the inflammatory response in obese AT seems to be preferable. An intriguing approach with clear and rapid translational potential is the use of Food and Drug Administration (FDA)-approved drugs, e.g., statins.

Statins are lipid-lowering drugs that inhibit HMG-CoA reductase, which plays a central role in the production of cholesterol. High cholesterol levels have been associated with cardiovascular disease (CVD). Statins have been found to reduce CVD and mortality in those who are at high risk [122]. The evidence is strong that statins are effective for treating CVD in the early stages of a disease (secondary prevention) and in those at elevated risk but without CVD (primary prevention). There is intense interest in repurposing statins in cancer [123]. A recent meta-analysis of 95 cohorts including 1,111,407 individuals concluded that statin therapy has potential survival benefit for patients with malignancy [124]. Several large studies support the preventive effect of statins in selected cancers. Although some early epidemiologic studies reported no beneficial effects of statins on PDAC risk [125,126,127,128], several recent studies associated statins with a significantly lower risk of cancer, including PDAC [129,130,131,132,133,134]. In a large case-control study, statin use was associated with a 34% reduced PDAC risk, with a stronger association in male subjects [133]. In a case control study of 408 patients with PDAC and 816 matched controls, statin use was associated with reduced PDAC risk (odds ratio: 0.61; 95% CI: 0.43–0.88) [135]. In this study, the protective effect of statin was dose-dependent and stronger in obese patients. Very recently, a meta-analysis of 26 studies containing more than 3 million participants and 170,000 PDAC patients found a significant decrease in PDAC risk with statin use (RR: 0.84; 95% CI: 0.73–0.97) [136]. In patients with chronic pancreatitis, statins lowered the risk of progression and pancreatic cancer [137]. In addition, statins have been shown to improve survival after resection of early PDAC, indicating a potential benefit for secondary prevention [138,139,140].

**Figure 1:**
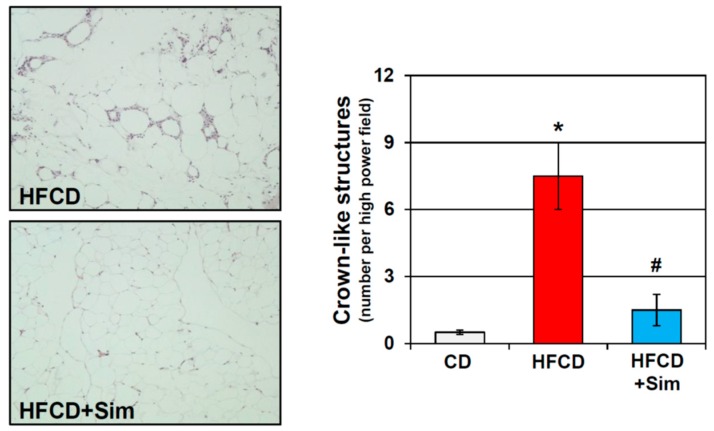
Representative histology of mesenteric adipose tissue of conditional KrasG12D mice fed an obesogenic high fat high calorie diet (HFCD) for 3 months supplemented without (upper panel) or with simvastatin (sim; lower panel). Quantification of crown-like structures demonstrates significant elevation of adipose tissue inflammation in obese HFCD-fed mice, which was abrogated by simvastatin (sim). *: *p* < 0.01 vs. CD; #: *p* < 0.01 vs. HFCD

Besides their cholesterol lowering effects, statins are known to have anti-inflammatory properties [141]. Statins attenuate AT inflammation in various human conditions and experimental models [142,143,144,145]. The improvement of AT inflammation by statins correlated with a reduction of inflammatory cell infiltration. In addition, statins have been found to decrease circulating MCP-1 levels, which are elevated in obese patients [76,146]. Importantly, statins elicited an anti-inflammatory M_2_-polarization of macrophages [145,147]. In our own study using a genetically engineered mouse model of pancreatic cancer promoted by diet-induced obesity, we found that oral administration of simvastatin attenuated early pancreatic neoplastic progression [148], which was accompanied by a marked reduction of inflammation in the VAT (unpublished; Figure 1).

Mechanistically, statins inhibit 3-hydroxy-3-methlyglutaryl coenzyme (HMG-CoA) reductase, the rate-limiting enzyme that controls the conversion of HMG-CoA to mevalonic acid. Mevalonic acid is a precursor of isoprenoids, which are essential for the post-translational modification of small G-proteins, e.g., Ras homolog gene family, member A (RhoA), cell division control protein 42 homolog (Cdc42), and Ras-related C3 botulinum toxin substrate 1 (Rac1), to attach to lipid membranes. Activation of Rho family members GTPases (guanosine triphosphate hydrolases) modulate the actin cytoskeleton, which has been shown to be an important regulator for macrophage polarization [149]. Our own unpublished data showed that lipophilic statins significantly attenuate pro-inflammatory signaling in macrophage cell lines through inhibition of the mevalonic acid/protein prenylation/actin cytoskeleton pathway. Besides their beneficial effects on AT inflammation, statins also have significant effects on PDAC cells [150,151,152]. Overall, statins show great promise for PDAC prevention/interception, in particular in the setting of obesity.

## 4. Conclusions

There is clear evidence that obesity increases the risk of pancreatic cancer. Obesity-induced AT inflammation is increasingly recognized as an important driver of the pathophysiologic process underlying the obesity–PDAC link. Dramatic changes in immune cell number and function in obese AT, especially VAT, with accompanying increased production of various pro-inflammatory cytokines may thereby promote the proliferation and survival of transformed cells in the adjacent pancreas. Although numerous immune cells have been shown to be involved in maintaining sustained AT inflammation in obesity, AT macrophages clearly play a central and critical role in orchestrating the inflammatory response. Given the importance of AT inflammation as a strong promotional driver of obesity-associated PDAC, strategies to inhibit obesity-induced AT inflammation are an intriguing approach. In this regard, the re-purposing of FDA-approved drugs aimed at reducing obesity-induced AT inflammation holds great translational significance. Recent epidemiologic data and preclinical evidence strongly suggest a role of statins, especially lipophilic statins, as promising drugs for PDAC prevention/interception. Besides their direct effects on PDAC cells, current data suggest that the anti-cancer properties of statins might at least partially include their anti-inflammatory effects on obese AT.
